# Development of a Virtual Reality Simulator for an Intelligent Robotic System Used in Ankle Rehabilitation

**DOI:** 10.3390/s21041537

**Published:** 2021-02-23

**Authors:** Florin Covaciu, Adrian Pisla, Anca-Elena Iordan

**Affiliations:** 1 Department of Design Engineering and Robotics, Technical University of Cluj-Napoca, 400641 Cluj-Napoca, Romania; florin.covaciu@muri.utcluj.ro (F.C.); adrian.pisla@muri.utcluj.ro (A.P.); 2 Department of Electrical Engineering and Industrial Informatics, Polytechnic University Timisoara, 331128 Hunedoara, Romania

**Keywords:** sensors, virtual reality, machine learning, ankle rehabilitation, simulator, intelligent robotic system

## Abstract

The traditional systems used in the physiotherapy rehabilitation process are evolving towards more advanced systems that use virtual reality (VR) environments so that the patient in the rehabilitation process can perform various exercises in an interactive way, thus improving the patient’s motivation and reducing the therapist’s work. The paper presents a VR simulator for an intelligent robotic system of physiotherapeutic rehabilitation of the ankle of a person who has had a stroke. This simulator can interact with a real human subject by attaching a sensor that contains a gyroscope and accelerometer to identify the position and acceleration of foot movement on three axes. An electromyography (EMG) sensor is also attached to the patient’s leg muscles to measure muscle activity because a patient who is in a worse condition has weaker muscle activity. The data collected from the sensors are taken by an intelligent module that uses machine learning to create new levels of exercise and control of the robotic rehabilitation structure of the virtual environment. Starting from these objectives, the virtual reality simulator created will have a low dependence on the therapist, this being the main improvement over other simulators already created for this purpose.

## 1. Introduction

In parallel with the achievements in engineering in recent years, medicine has evolved a lot, but there are still unresolved problems. A rather important problem remains stroke in both its forms (ischemic and hemorrhagic) which is considered the leading cause of disability and the second leading cause of death worldwide [1], becoming a growing problem of contemporary society and any progress made in treating this disease is a step forward in the fight against this disease. Recovery after a stroke is different for each person, depending on the condition of each, and to make a successful recovery in both forms of stroke, treatment will often involve specific therapies and support, such as: speech therapy, occupational therapy, physical recovery post-AVC, support groups, support from friends and family [2]. Rehabilitation after a stroke should be done as soon as possible with intensive and repetitive exercises, although this may reduce the patient’s motivation. One way to motivate and help patients regain their motoric skills is to introduce virtual reality technology [3].

This article presents a simulator using virtual reality for a robotic system that is controlled by an intelligent module that uses machine learning to optimize the ankle recovery treatment of a stroke patients, using visual stimulation in the recovery process. The patient is in real-time interaction with the system via a special sensor device that is attached to the patient’s limb. On this device the sensors are connected to a microcontroller that contains a client application through which it sends data collected from the sensors via the Wi-Fi connection to a server application that is on the computer via the TCP/IP protocol, and this data is retrieved and processed by the intelligent module. This module uses a machine learning algorithm that will result in the type of exercise that the patient will perform and the level of difficulty of this exercise. The real person will be able to do various ankle rehabilitation exercises by interacting with the VR simulator. This simulator can be used in the home of any patient who has had a stroke, without incurring additional costs. Usually, a rehabilitation process takes place in specialized physiotherapy centres where a professional in the area supervises or helps.

A recent clinical study involving a number of 23 patients with different neuro-motor impairments demonstrated that following a 7-day rehabilitation program applied at the level of the upper limb, no significant differences were identified between the robotic assisted and the manual therapy. Taking into account that the robotic system used in this experiment is still in development the results are supporting the idea of using robotic systems in rehabilitation illustrating also the potential advantages that additional, support technologies could improve the outcome of physical therapy [4]. Moreover, the engagement of patients in the therapy could bring benefits for the rehabilitation procedures. In other words, if the patient is not bored and is actively engaged in the exercises (e.g., by playing games in an interactive way) the rehabilitation may have better outcomes by improving the patient’s motivation and reducing the work of the therapist [5].

This article is structured as follows: after introduction, the next section reviews the current state of the art, continuing with its robotic system architecture in Section 3. Section 4 presents development of the software application followed in Section 5 by conclusions, acknowledgments, and references. Compared to other rehabilitation systems, the robotic rehabilitation system presented in this paper has as a novelty the use of an intelligent module implemented using KNN. This intelligent module, based on the data provided by the sensors and the user’s previous results, can determine without the intervention of the therapist the level of difficulty of the exercise to be performed by the user.

## 2. Background

Various physiotherapeutic rehabilitation systems can be found in the current literature that are used for people who have had a stroke In recent years these systems have evolved into more advanced systems that include virtual reality (VR) environments being used to improve rehabilitation techniques and physical executions.

A recent example is the system called EXOMedical (University of Salamanca, Salamanca, Spain) which includes an exoskeleton for the elbow. This exoskeleton has an axis of rotation corresponding to the axis of rotation of the elbow joint and consists of two parts that are attached to the outside of the arm and forearm with a series of Velcro straps type, being a low-cost exoskeleton because in its construction was used 3D printer technology. This rehabilitation system uses VR technology so that the patient in the rehabilitation process can perform various exercises in an interactive way improving the patient’s motivation and reducing the work of the therapist. The user using this system views an avatar that represents his movements in the virtual environment with the ability to recognize if he has completed the task received. The system can automatically change some of the task parameters, according to the real-time analysis provided by the Unity virtual reality environment control unit, making it possible to modulate each difficulty level of the exercise or the maximum time to complete the exercise [5].

Scalona et al., conducted a study evaluating muscle synergies when seventeen healthy subjects perform throwing tasks in both a virtual and a real environment. For each subject in the study were recorded electromyographic (EMG) signals of 11 upper limb muscles. At the end of the study, encouraging results were found for the application of virtual reality to complete the conventional therapy in the rehabilitation process [6].

Sierotowicz et al., described a system called BodyRig, which has the ability to track a user’s body posture in real time, both in VR and in real life. The potential of this system is not limited to a robotic rehabilitation system. The performance of this system is evaluated in an online task of tracking the trajectory in VR using the device through an experiment involving 10 subjects, showing that an average user can reach an accuracy of 0.66 cm during a static precision task and 6.33 cm while following a moving trajectory when tested in a user’s complete personal space [7].

Luzio et al., show that intensive and repetitive exercises during robot-assisted rehabilitation can expose patients to an inappropriate and unsafe position, and in this regard the authors introduced sensory and visual feedback in a robotic system for upper limb rehabilitation to provide information about the incorrect posture of the neck and torso. For the study, 10 healthy subjects were used, each of them performing 3D touch movements using the robotic platform in three different conditions, meaning with visual feedback, with vibrotactile feedback and without feedback, after which a comparative analysis was performed to evaluate performance. The experimental result showed that if there is no feedback the subjects do not maintain their correct body position, and instead with visual or vibrotactile feedback the subjects tend to correct the inappropriate posture during the execution of the task [8].

Joo et al., shows the effect of VR in the rehabilitation of a burned hand. In this study, the authors used 57 patients with burned-out to compare the rehabilitation process using exercises with VR technology and conventional rehabilitation where VR is not used. To use VR technology in the rehabilitation process on the hands some gloves called RAPAEL Smart Glove were used, with which patients had to do various exercises. The results of the study suggested that VR-based rehabilitation is as effective as conventional rehabilitation for recovering the function of a burned hand [9].

Ferreira et al., developed a game based on VR that is played by patients in different stages of recovery of the upper limbs, motivating patients to continue performing exercises beyond what is usual with conventional techniques [10].

Zakharov et al., tested a combination of virtual and robotic reality for 10 days of 15 min each in restoring gait for a person who had an acute stroke using a visual and tactile biofeedback simulation based on virtual reality immersion and physical impact on patients’ soles. At the end of this study, an improvement in the motor function of the lower extremities was found [11].

Wang et al., presents a virtual reality simulator for a lower limb rehabilitation robot that can simulate cycling, encouraging patients to join recovery training through a built-in competitive game. The synchronization of the movement between the robot and the virtual model is achieved through an interaction control strategy, the robot being able to modify the training speed based on the feedback signal of the field in game. This training can be paused by the patient and the doctor at any time, and the timer function could reflect the patient’s recovery [12].

Lee et al., performed a meta-analysis to examine whether virtual reality training is effective for both lower and upper limb function and general function in patients with chronic stroke. As a conclusion, virtual reality training has been effective in improving function in patients with chronic stroke, corresponding to a moderate effect size. Virtual reality training has also shown a similar effect in improving upper and lower limb functions [13].

Mirelman et al., conducted a study involving 15 men and three women with chronic hemiparesis after a stroke. Two groups were formed for this study. The first group trained on the Rutgers ankle rehabilitation system without being connected to virtual reality. The training was performed 3 times a week for 4 weeks for 1 h at each visit. Subjects performed ankle movements in dorsiflexion, plantar flexion, inversion, eversion, and a combination of these movements. At the beginning of each session, strength, speed and performance were measured and then used as references for the following exercises. Subjects in the second group who trained with the Rutgers ankle rehabilitation robot using virtual reality performed exercises using foot movements to navigate an airplane or boat using the virtual reality environment that contained a series of targets. To ensure training, the position and timing of the targets were manipulated, including discrete and combined ankle movements. Subjects who trained only with the robotic system without being connected to virtual reality received the same exercises as the group who trained with the robotic system with virtual reality, but without the virtual environment. Subjects in the robotic system group who did not use virtual reality were instructed by a therapist on the direction in which to move their leg and the number of repetitions to be comparable to the group that used the robotic system with virtual reality. Feedback for the group that used virtual reality was provided by the simulation consisting of knowledge of performance and knowledge of results. At the end of the training, the subjects in the group with the robotics system without virtual reality reported fatigue earlier in the sessions compared to the subjects in the group who used the robotics system with virtual reality. There have also been reports of clinically significant improvements and gait improvements in subjects with chronic hemiparesis who have been trained for 4 weeks with the robotic ankle rehabilitation system that is coupled with virtual reality [14].

Burdea et al., made a study to feasibility of game-based robotic training of the ankle in children with cerebral palsy. This study was done for 12 months in a university research laboratory. The subjects were three children with cerebral palsy, aged between 7 and 12 years, all male. They were trained for 36 rehabilitation sessions for 12 weeks using the Rutgers Ankle CP system (Rutgers, the State University of New Jersey, New Brunswick, NJ, USA) playing two types of virtual reality games. During the game, the subjects were seated, and trained one ankle at-a-time for strength, motor control and coordination. At the end of the session, the results indicated varying degrees of improvement in ankle endurance, gait and speed [15].

Zimmerli et al., investigate the influence of different design characteristics of virtual reality exercices on engagement during lower extremity motor rehabilitation. This study involved 10 subjects without any neurological movement disorder and 12 subjects with spinal cord injuries (SCI), and this study was conducted at the Paraplegic Center of Balgrist University Hospital (Zurich, Switzerland). The inclusion criteria for subjects with SCI was to be able to stand upright for at least 30 s, with or without support. Subjects with chronic post-injury greater than one year were also included, as well as subjects with SCI with acute post-injury less than 1 year. Excluded were those who showed signs of depression, skin lesions in the lower limbs, osteoporosis, cardiovascular instability, uncontrolled spasticity that would significantly interfere with the movement of the lower extremities, acute medical conditions, those who had a higher height of 190 cm or weighing more than 135 kg. To verify the involvement of the subjects, four types of virtual reality exercises were created and designed in front of the subjects, while they were introduced in the robotic orthosis system Lokomat. The exercises performed were at constant speed, higher speed, sprint and race. During the exercises, the heart rate and electromyographic activity for biceps femoris were recorded. The study showed that functional feedback is extremely important for the active participation of patients during robotic rehabilitation care [16].

Esfahlani et al., presents a robotic system called “ReHabGame” which is a serious game using a fusion of implemented technologies that can be easily used by patients and therapists to evaluate and improve sensorimotor performance. This system allows a subject to control the avatar’s movements through a Kinect Xbox sensor (Microsoft, Redmond, Washington, WA, USA), Myo armband (Thalmic Labs, Kitchener, ON, Canada) and a rubber foot pedal. Data is collected from sensors and taken over by a fuzzy system to create new levels of difficulty. This system addresses the kinematic activity of the upper and lower limbs which, at the same time, provides requirements to meet the tasks according to the needs of the patient [17].

Based on the current state of the art it was concluded that the difficulty of improving these systems is the development of an intelligent module with VR that should help in the rehabilitation process by replacing a significant number of tasks that were otherwise performed by the therapist. This in turn enables the therapist to work with multiple patients at one time.

## 3. Robotic System Architecture

This section presents the architecture of the proposed system. At the beginning, a description is made of the interconnection of the components of the intelligent robotic system. Next, the mechanical rehabilitation structure of the robotic system is described, and at the end a description of the user interface is made.

### 3.1. Components and Connections in the Robotic System

**Figure 1:**
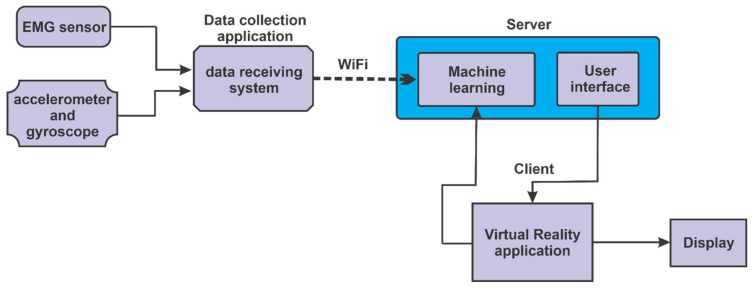
Interconnection of components.

In the block diagram from Figure 1 an interconnection of the components of the logic in the control of the intelligent robotic system can be seen. Data of the muscle state of the lower limb is obtained via electromyography sensor (EMG), the accelerometer sensor retrieves data about acceleration made by the lower limb during movement, and the gyroscope sensor retrieves data on the position of the lower limb. This data, after being collected by the data collection application and using Wi-Fi connection, will be taken over by the server application which will send this data to the intelligent learning system. After the data is processed by the intelligent learning system, and the level of difficulty as well as the degree of help of the patient will be predicted, this system will send data to the client application, which contains the virtual reality application, and this virtual reality application can be viewed on a display. Monitoring the progress of the levels of exercises that the patient does is done by the intelligent system, the client application (VR) sends data related to the progress to the intelligent learning system.

**Figure 2:**
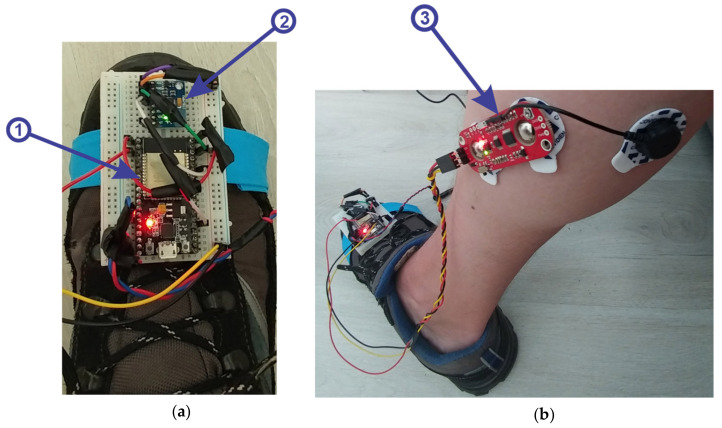
(**a**) Device for retrieving and sending data from sensors to the Server application. (**b**) MyoWare—electromyography sensor

For the client application that is used to retrieve data from the sensors, a device has been created that attaches to the patient limb at the sole, as can be seen in Figure 2. This device contains the following components:

(1) microcontroller ESP32 (Figure 2a, 1), is a strong development board, containing the following:
  - Wi-Fi, Bluetooth and a dual-core processor; frequency: 2.4~2.5 GHz; power supply: 7~3.6 V; size: 18 mm × 25.5 mm × 3.1 mm.
(2) 3-axis accelerometer and gyroscope module, model MPU6050, with the following specifications:
  - supply voltage: 3.3–5 V (LDO regulator included); I^2^C bus voltage: 3.3 V (MAX); current: 5 mA; programmable gyroscope range: ±250, ±500, ±1000, ±2000 o/s; programmable accelerometer range: ±2 g, ±4 g, ±8 g, ±16 g; maximum I^2^C frequency: 400 kHz.
(3) muscle sensor, MyoWare model with the following specifications:
  - single supply, +2.9 V to +5.7 V with polarity reversal protection; two output modes: EMG Envelope and Raw EMG; LED indicators; adjustable gain.

### 3.2. Description of the Main Components of the Robotic Rehabilitation Structure

The robotic rehabilitation structure was designed in the Fusion360 program [18]. This program is a cloud-based CAD, CAM and CAE 3D design tool from Autodesk (San Rafael, California, CA, USA), a complete program available to enthusiasts and start-up companies at no cost.

**Figure 3:**
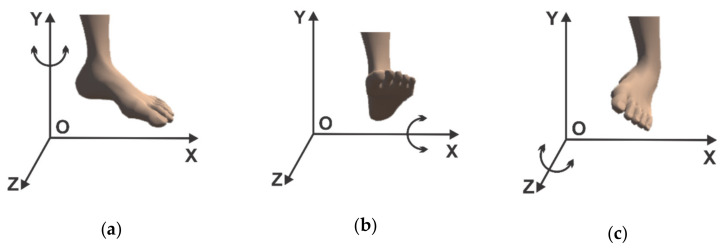
(**a)** Ankle movement by rotation around the Oy axis. (**b**) Ankle movement by rotation around Ox axis. (**c**) Ankle movement by rotation around Oz axis

The robotic structure that was designed in this program has three degrees of freedom, and the possible movements for the rehabilitation of a patient’s ankle with the help of this structure are the following:

(1) rotation in horizontal plane (parallel to the xOz plane) around the Oy axis (Figure 3a);
(2) rotational motion in vertical plane (parallel to the yOz plane) around the Ox axis (Figure 3b);
(3) the third motion shown consists of a rotation around the Oz axis (in a plane parallel to the xOy plane) (Figure 3c).

**Figure 4:**
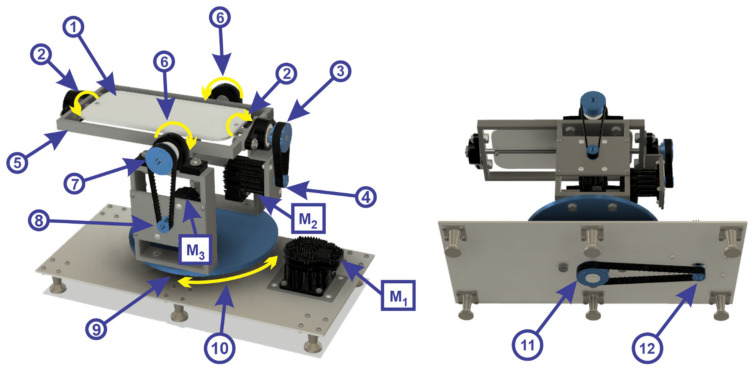
Robotic rehabilitation structure.

In order to perform rehabilitation exercises with the help of the robotic structure, the foot must be positioned on the mobile platform in Figure 4 as follows:

- mobile platform 1 rotates about the Oz axis in a counterclockwise and clockwise direction, by means of a toothed wheel (2);
- toothed wheel (2) is driven by a transmission belt (3) by means of a toothed wheel (4);
- toothed wheel (4) is driven by a servomotor (M_2_) from TEKNIC, model CPM-SDHP-2311S-ELS;
- the mobile platform 2 (5) rotates vertically plane around the Ox axis in a counterclockwise and clockwise direction (6) by means of a toothed wheel (7);
- toothed wheel (7) is driven by a drive belt by means of a toothed wheel (8) being driven by a servomotor (M_3_) from TEKNIC, model CPM-SDHP-2311S-ELS;
- the mobile platform 3 (9) make a rotation in horizontal plane (parallel to the xOz plane) around the Oy axis in the counterclockwise and clockwise direction (10) by means of a toothed wheel (11);
- toothed wheel (11) is driven by a transmission belt by means of a toothed wheel (12) being driven by a servomotor (M_1_) from the company TEKNIC, model CPM SDHP-3411S-ELS.

### 3.3. Description of the Graphical User Interface

**Figure 5:**
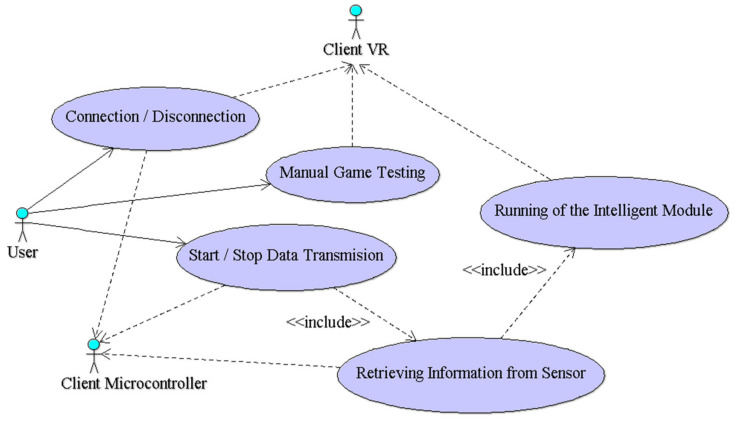
Use case diagram corresponding to the server application.

The software application architecture includes the server application and two client applications: one associated with the microcontroller to retrieve information from the sensors, and the other for the virtual reality application. In the analysis phase, for a clear specification of the server application, the use case diagram [19] is made (Figure 5) using UML [20].

**Figure 6:**
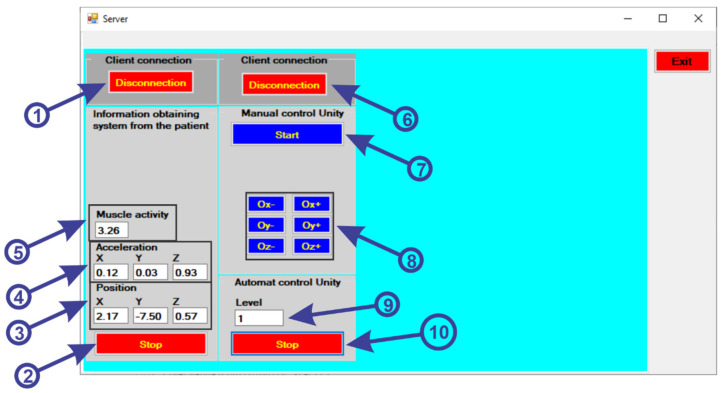
User interface.

Starting from the functionalities specified in the use case diagram, the graphical user interface of the server application was developed using the C # programming language [21] and the Microsoft Visual Studio development environment [22]. In order to be able to communicate between the data collection system from the sensors, the intelligent module and the virtual reality, a user interface is used (Figure 6). To use the user interface, the following steps will be followed:

- first the “ConnectESP32” button (1) is pressed to make the connection via Wi-Fi connection between the Server application and the Client application located on the ESP32 microcontroller;
- to receive the data from the sensors, we must press the “Start” button (2);
- after pressing the button, the data is collected and displayed from the gyroscope sensor (3) for position, accelerometer (4) to identify the acceleration and the muscle activity sensor (5) to identify the state of muscle tone.
- to make the connection between the server and the client application of the virtual reality application, the “ConnectUnity” button must be pressed (6);
- after the communication has been established, we must press the “Start” button (7) to start the communication for the manual control of the virtual reality application, without including the intelligent module;
- using the following buttons (8), a test of the robotic rehabilitation structure is performed to perform the various exercises, as follows:
  - when we press the “Ox−” button a clockwise rotation is made around the Ox axis;
  - when we press the “Ox+” button a counterclockwise rotation is made around the Ox axis;
  - when we press the “Oy−” button a clockwise rotation is made around the Oy axis;
  - when we press the “Oy+” button a counterclockwise rotation is made around the Oy axis;
  - when we press the “Oz−” button a clockwise rotation is made around the Oz axis;
  - when we press the “Oz+” button a counterclockwise rotation is made around the Oz axis;
- by pressing the “Start” button (10) the virtual reality application is started automatically, the application communicating with the intelligent module to create the range of levels for the virtual reality application (9).

## 4. Software Application Development

### 4.1. Description of the Operation of the Virtual Reality Application

**Figure 7:**
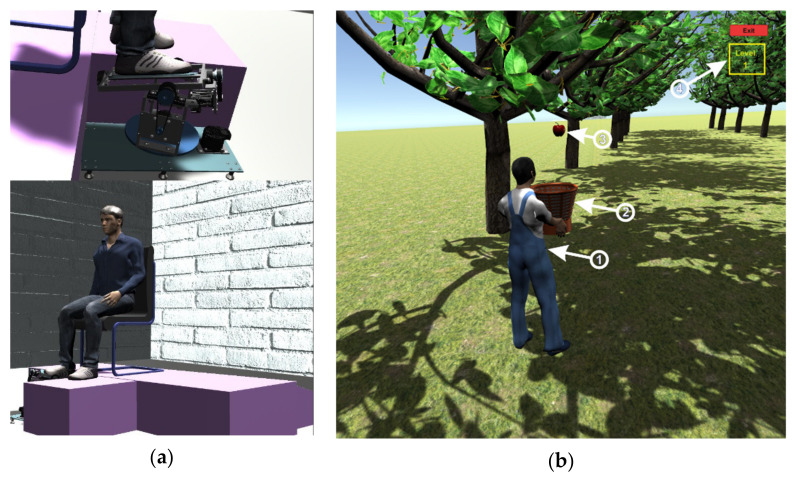
(**a**) Virtual patient who performs rehabilitation exercises with robotic structure. (**b**) Virtual human character picking apples

Before starting the virtual reality application, the system for retrieving data from the sensors is attached on the foot of a real human subject and an EMG sensor is attached to the foot muscles, as can be seen in Figure 2. When starting the virtual reality application, first run the server application by pressing the “ConnectESP32” button to make the connection between the server application and the client application located on the microcontroller (Figure 6, 1), then click on the “Start” button to start the client application to receive data from sensors (Figure 6, 2), these data being taken over by the intelligent module and processed. The next step is to connect between the server and the virtual reality application which is a client application, by pressing the “ConnectUnity” button (Figure 6, 6). Once the connection with the virtual reality application has been made, it is necessary to press the “Start” button (Figure 6, 10) to start the communication between the intelligent module and the virtual reality application. At this point the real human subject must look at the computer monitor while running the virtual reality application (Figure 7) and identify the falling apples (Figure 7b, 3) in order to catch them in the basket (Figure 7b, 2) using the human virtual character (Figure 7b, 1). To catch apples, the real human subject must make different movements of the foot paw to position and move the human virtual character in order to collect the apples. In this process the real human subject performs different ankle rehabilitation exercises, and all this time in the virtual reality application the robotic rehabilitation structure makes different rehabilitation movements for the human virtual character, as can be seen in Figure 7a). During the control of the human virtual character who has to pick apples, the following movements must be made, as follows:

- to control the walking of the human virtual character, the patient must make a rotation in vertical plane of the ankle (parallel to the yOz plane) around the Ox axis (Figure 3b). By rotating the ankle around the Ox axis in the counterclockwise direction, the human virtual character goes slowly, and by rotating the ankle around the Ox axis in the clockwise direction, the human virtual character begins to run;
- to direct the human virtual character as it goes left and right, the real human subject must make a rotational movement of the ankle in a horizontal plane (parallel to the xOz plane) around the Oy axis (Figure 3a). When a rotation movement is made in a counterclockwise direction, the human virtual character turns to the left, and when a rotation is made in a clockwise direction, the human virtual character turns to the right;
- in order to make a 180-degree rotation of the human virtual character, the patient must make a rotation of the ankle around the Oz axis (in a plane parallel to the xOy plane) (Figure 3c) in counterclockwise and clockwise direction.

The intelligent module receives information from sensors (Figure 2) on the condition of the ankle and from the virtual reality application on the performance of the human virtual character in picking apples (Figure 7b). Depending on the performance of the real human subject in performing exercises that train the human virtual character to pick apples, the intelligent module creates new levels of exercises (Figure 7b, 4) and levels of control of the virtual robotic structure (Figure 7a).

### 4.2. Intelligent Module Description

Machine learning [23], according to the specialized literature, includes both unsupervised learning [24] and supervised learning. Supervised learning [25] is based on a training model that uses a set of labeled data. This model is then used to map between a series of new observations and their category based on a function that generates it at the time of training. There are two types of supervised learning: classification [26] and regression [27].

#### 4.2.1. K-Nearest Neighbours

For the project presented in this paper, supervised learning based on classification was used because the data set used for learning is a labeled one, and the labels are discrete values. The presented project aims to automate the process of determining the level of difficulty, as well as the help given by the platform to the patient to perform the tasks in the rehabilitation game. Since there are 5 levels of difficulty for the rehabilitation game, and the platform will be able to help the patient in 4 steps for each level, the machine learning algorithm will have to be a classifier that will have an output that will fall into one of the 20 possible classes (20 = 5 × 4).

The classification algorithm, as a supervised learning technique, is provided with both the instances and their labels, and in the processing phase the algorithm must find patterns between the instances and their classes, so that when they have to classify a new instance to may decide, on the basis of the instances already classified, the class to which the new instance belongs.

**Figure 8:**
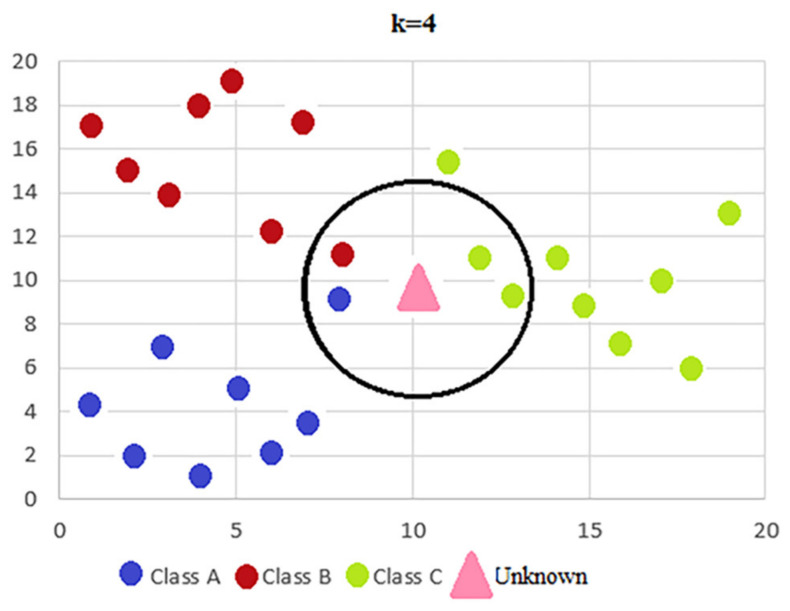
Graphical representation of KNN algorithm.

There are several classification techniques such as: K-nearest neighbors [28], decision trees [29], random forest [30] and feed-forward neural network [31]. This project will use the K-nearest neighbors (KNN) algorithm which is based on a similarity of characteristics, which means that the level of similarity of the instances characteristics with those of the training set determines how a new observation will be classified. A new set of observations is classified based on the characteristics of its neighbors, the class being determined by the majority among its neighbors. In order to be able to carry out this process, KNN must keep in memory the entire data set required at the time of classification, as there is no prior training process. The process of selecting neighbors and determining the final class of an instance is represented graphically in Figure 8.

#### 4.2.2. Preliminary Analysis and Preprocessing of the Data Set

To obtain data from the EMG sensor for the detection of electromyographic activity (EMG), surface electrodes are fixed on the skin covering the muscle, forming a charge layer at the interface between the metal electrode and an electrolyte solution. During the measurement with this sensor there are two sources of motion artifact in the surface electrodes, namely: mechanical disturbance of the charging layer of the electrode and deformation of the skin under the electrodes. In order to have a more accurate measurement with this sensor it is necessary to use a filter that filters these disturbances. We chose to use the Kalman filter on the microcontroller. This filter is an algorithm that provides estimates of unknown variables given the measurements observed over time. To calibrate the Kalman filter algorithm, raw voltage data samples were first read and taken from the EMG sensor during operation, after which these data were entered in an Excel table to determine the variance. After the variance was determined, the variance value obtained was entered as a variable (R) in the Kalman filter algorithm. For the process variation variable (Q) a value was set which was subsequently adjusted by experimentation to obtain the desired filter performance.

When using the MPU6050 sensor which includes the gyroscope and accelerometer due to the influence of semiconductor thermal noise and electromagnetic interference the output has a certain noise that affects the signal accuracy, thus interfering with the accuracy of the stability of the entire system. In order to obtain more accurate data during the operation of the MPU6050 sensor we chose to filter the data using the Kalman filter. This filter not only can filter the signal coming from the gyroscope, but also can filter the signal coming from the accelerometer [32]. To filter the signal from the gyroscope and accelerometer we implemented a program with the Kalman algorithm on the microcontroller that takes data from these sensors, and to implement the Kalman algorithm we used the steps we described in implementing the EMG sensor algorithm.

The data set is a collection of labeled observations that define certain characteristics of an instance with respect to the rehabilitation exercises. Based on such a collection of observations, learning algorithms are used to predict the labels of instances with similar characteristics to the collection. An example of the input data set is shown in Table 1. The format of the input data set has the following structure:

- M—the value returned by the sensor that measures muscle intensity;
- Ax—projection on the Ox axis of the measured acceleration;
- Ay—projection on the Oy axis of the measured acceleration;
- Az—projection on the Oz axis of the measured acceleration;
- Px—projection on the Ox axis of foot position;
- Py—projection on the Oy axis of foot position;
- Pz—projection on the Oz axis of foot position;
- S—value that specifies the score obtained by the patient in the previous game.

**Table 1:** The format of the input data.

M	Ax	Ay	Az	Px	Py	Pz	S
0.0774	−0.0498	0.0007	0.9277	248.554	1297.854	1765.564	0
0.0105	−0.1013	0.0378	0.9338	246.6993	1284.16	1777.141	0.4
0.2208	−0.1143	0.2205	0.8694	256.5768	1259.951	1792.288	0.8
0.7986	−0.1304	0.1609	0.885	243.2331	1246.401	1810.49	0.6
0.9203	−0.1643	0.1575	0.8877	250.1196	1231.634	1817.76	1

For the performance of a classification model to be optimal, it must have a well-structured data set and a large number of instances. Labels are the classes in which a measurement can be classified, and for this data set the number of classes is 20. Each measurement will belong to one of these classes.

**Figure 9:**
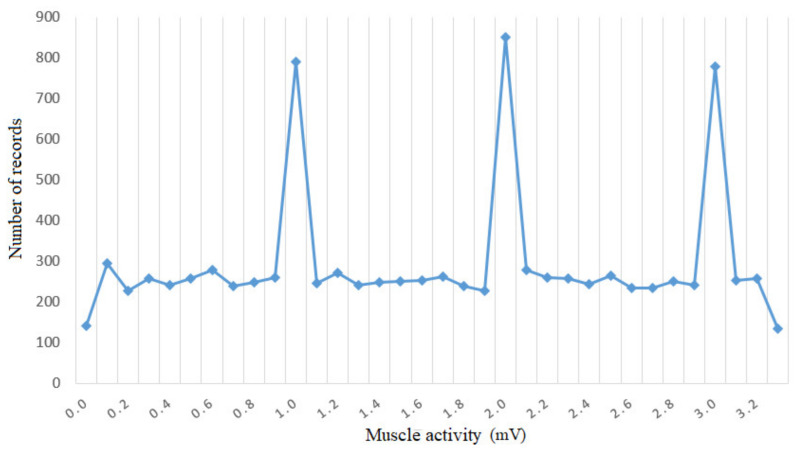
Histogram for muscle activity.

In order to analyze the data set, a histogram was generated (Figure 9) representing the number of instances with the same value of muscle intensity in the data set. The figure shows that there are values of muscle intensity that predominate in the data set and there are others that have a small number of occurrences. It follows that the data set has an imbalance between the labeled instances, as they are labels that predominate considerably in the data set and labels that have a very small number of instances compared to the majority classes. In conclusion, a data preprocessing process is required by using a data set balancing technique.

**Figure 10:**
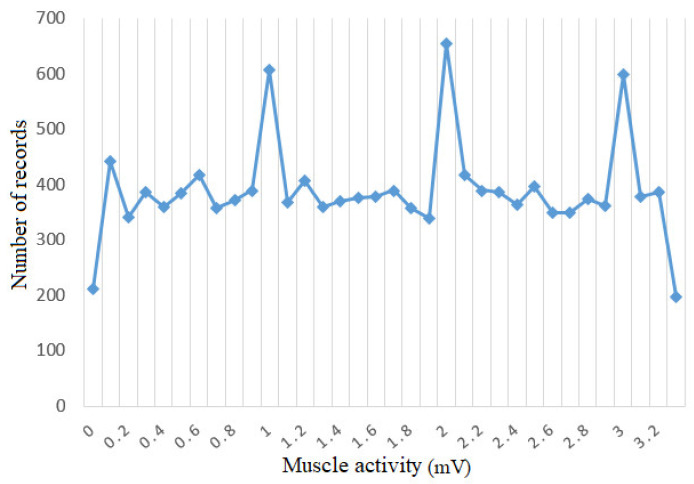
Histogram for muscle activity after balancing.

An optimal solution is to use the oversampling method followed by the application of the undersampling method to the result of the first method. The purpose of applying the two methods is to eliminate the overfitting of the data set created by oversampling. The undersampling method [33] involves reducing the number of records in the majority classes by random deletion or by certain established criteria until the data set becomes balanced. The oversampling method [34] is the opposite of the previous method, involving the generation of artificial data using minority classes and replicating the data in order to balance the data set. This method has a major advantage over the other method because it does not lose the existing information, but, through replication, the overfitting effect can occur. Following the combination of the 2 methods, the data distribution is balanced. Figure 10 shows how most instances regardless of label are distributed approximately evenly between classes. Thus, for the KNN model, the data set will be easier to learn and easier to predict new instances provided to the model.

#### 4.2.3. Model Training

An important decision in the design and implementation of the solution is represented by the establishment of the data set for training, testing and validation. For the training data set we kept 75% of the initial data set, for the test data set we kept 15% of the initial data set, and for the validation data set the remaining 10%. Before being distributed between the three sets, we randomly shuffled the data to preserve the proportions of class distribution and to avoid consecutive instances with similar attributes that could affect model performance.

**Figure 11:**
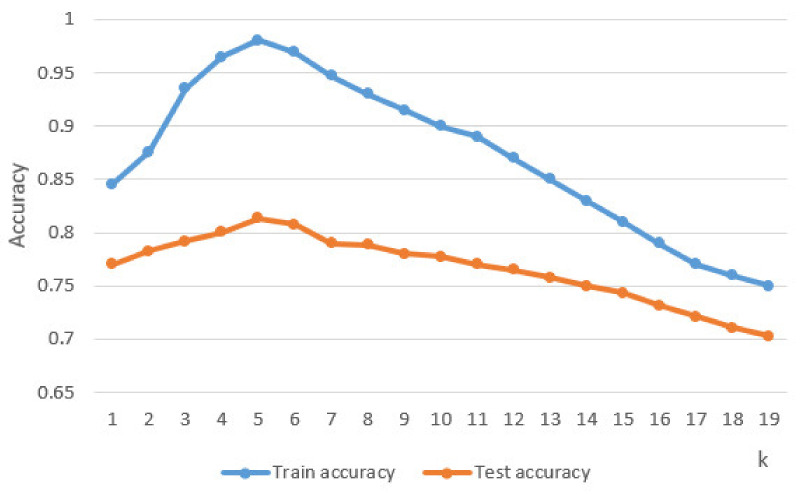
k tuning.

After implementing the KNN classifier in Python [35] using the TensoFlow library [36], its performance was tested based on the test set by determining the accuracy based on the classes predicted by it compared to the real classes of the instances. An accuracy of 81.35% was obtained, which is a fairly high accuracy that can be improved by parameter tuning [37]. For the KNN classifier the parameter that has been adjusted to determine maximum performance is k. This parameter represents the number of neighbors considered to determine classes for new unlabeled data instances. Thus, the instance to be classified is compared with its k neighbors and the final label for the new instance is determined. Figure 11 shows the accuracy of the classifier according to the parameter k. It can be seen that the best accuracy was obtained for k = 5.

#### 4.2.4. Evaluation of the Trained Model

**Figure 12:**
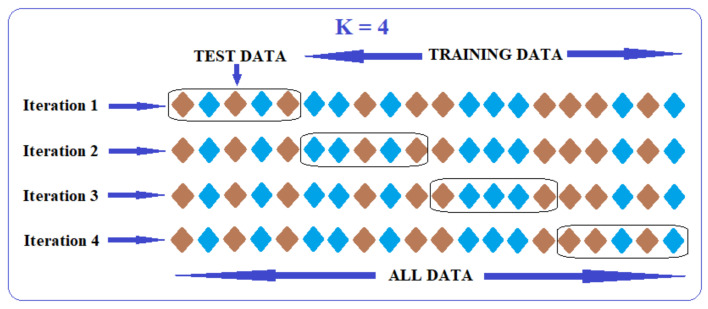
Graphical representation of K-fold cross-validation.

Among the existing models for the evaluation and validation of classifiers, the K-folds Cross-Validation method was used [38]. This method has as a principle the division of the data set into k complementary parts where k-1 of these parts are used for training and the last part is used for testing. This process is repeated of k times so that each subset of the data set is used for testing and the rest for training. Figure 12 shows both the functioning and data sharing in the K-folds Cross-Validation process [39,40]. This method can cause problems such as overfitting and can provide insight into how the system will evolve on new data.

**Figure 13:**
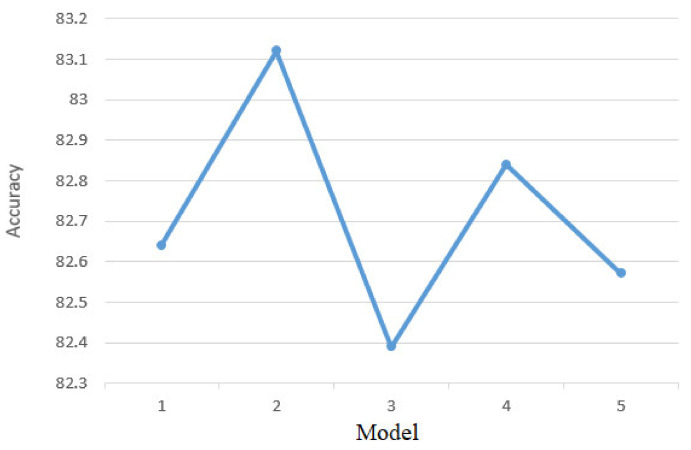
Five-fold cross-validation results.

To perform this validation phase, 5-fold cross-validation and 10-fold cross-validation were used, which involves two different validation processes. In the case of the first process the data were divided into five equal parts, and in the case of the second process in 10 equal parts. Each of these parts was used by turn for both model validation and training. After dividing the data into five parts, the accuracy of the KNN algorithm is represented in Figure 13. The model accuracy of 81.35% on the training data set and the accuracy after the Cross-Validation process are approximately equal which implies that the model does not have the problem of data overfitting. Because performances are approximately equal, it means that the model has generalized and found patterns in the dataset so that decisions are made based on the entire dataset and not on a particular dataset.

**Figure 14:**
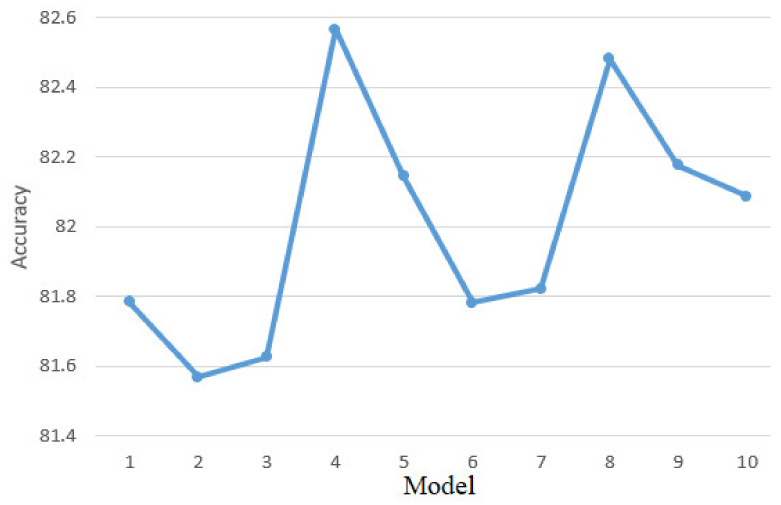
Ten-fold cross-validation results.

Figure 14 shows the performance of the KNN model for 10-fold cross-validation process. an analysis of the results leads to the same conclusions as in the case of 5-fold cross-validation process.

## 5. Conclusions

This article presents the development of a simulator for an intelligent robotic system designed to rehabilitate the ankle of a person who has had a stroke. In order to be able to make an interaction between the simulator and a real human subject, a sensor system is used that is attached to the real human subject. The simulator was developed in order to improve rehabilitation exercises through an interactive and motivating game for the people who use it. This simulator is able to detect muscle tone, position and acceleration of the lower limb to be rehabilitated during the exercises via the sensor system.

Compared to other rehabilitation systems that use virtual reality for interactive exercises, this system uses an intelligent module with machine learning to monitor patient progress and to establish the levels of exercise and a control of the robotic structure in the virtual environment. The KNN classification method was chosen for the implementation of the intelligent mode. The evaluation of the performances of the KNN algorithm was performed by balancing the data set used in the classification process, and by parameter tuning. Thus, an accuracy of the classification process of 81.35% was obtained. By using this intelligent module by the rehabilitation system, the patient’s recovery process can be improved, as he will no longer need the therapist at each rehabilitation session. As a future line of work, we want the physical construction of the robotic structure found in the virtual reality application and the use of more types of exercises for rehabilitation. At the same time, it is desired to implement other classification algorithms (Decision Trees, Random Forests) for the intelligent module and their comparison, so that the accuracy of the algorithm used in this rehabilitation system is as close as possible to 100%.

## Data Availability

Data sharing is not applicable to this article.
